# A techno-economic study on the utilisation of airborne wind energy for reverse osmosis seawater desalination

**DOI:** 10.1016/j.heliyon.2025.e41814

**Published:** 2025-01-08

**Authors:** Mahdi Ebrahimi Salari, Milad Zabihi, Jimmy Murphy

**Affiliations:** MaREI Centre, University College Cork, Cork, Ireland

**Keywords:** Airborne wind energy, Reverse osmosis, Seawater desalination, Energy recovery device, Levelised cost of water

## Abstract

Airborne wind energy is an emerging technology that can harness stronger and more consistent winds in higher altitudes using less mechanical and civil infrastructures than conventional wind energy systems. This article outlines a techno-economic study on using this technology for reverse osmosis seawater desalination in which a semi-permeable membrane process is used to remove salts and contaminants from water. To understand the techno-economic feasibility of such a system, this research work studies a 2 MW airborne wind-driven reverse osmosis plant. Different energy recovery devices are also studied to find their impact on improving the desalination plant's techno-economic performance. Results show the techno-economic practicality of an airborne wind-driven reverse osmosis plant with a competitive levelised-cost-of-water compared to similar-sized wind and solar energy-driven seawater desalination systems.

## Highlights

• A techno-economic analysis is performed on an AWE-driven RO system.
• Techno-economic impact of three different ERD devices is investigated.
• The techno-economic performance of the proposed system is compared to similar-sized wind and solar-powered RO systems.

**Table:** Abbreviations and nomenclature:


AWE	Airborne Wind Energy	PL	Pelton Turbine
CAPEX	Capital Expenditure	PV	Photovoltaic
CRF	Capital recovery factor	PX	Pressure Exchanger
EC	Energy Cost	RO	Reverse Osmosis
ERD	Energy Recovery Device	SEC	Specific Energy Consumption
FO	Forward osmosis	TWP	Total Water Production
HAWT	Horizontal Axis Wind Turbine	TEC	Total Energy Consumption
HTC	Hydraulic Turbine Charger	WACC	Weighted Average Cost of Capital
LCoE	Levelised Cost of Energy	PRO	Pressure retarded osmosis
LCOW	Levelised Cost of Water		
N	Lifetime of the system		
OPEX	Operating Expenditure		

## 1 Introduction

There has been growing attention towards using renewable energy in seawater desalination [[1], [2], [3]]. However, airborne wind energy has yet to be widely studied for desalination applications. The RO process removes salts and contaminants from water through a semi-permeable membrane. Among desalination methods, RO is more cost-effective and requires less energy [4]. RO systems also produce a high-pressure stream of the rejected salt water in addition to freshwater production. An energy recovery device (ERD) can use the rejected salt water to improve the system's efficiency. So far, several ERD technologies have been developed [4]. An ERD can improve the system's specific energy consumption (SEC) by using the recovered energy to feed the pumping station.

Hybrid desalination systems can improve efficiency by recovering energy from the rejected brine stream. One of the most famous hybrid membrane processes is pressure retarded osmosis (PRO). PRO is a novel technology that harnesses the natural process of osmosis to generate energy, particularly in the context of seawater desalination. The osmosis flow of water from the low-salinity water (feed stream) to high-salinity water (rejected brine from the RO system), which are separated by a semi-permeable membrane is used to generate energy. The pressure increase on the high salinity side due to the osmotic flow can be used to drive a turbine, converting the pressure into mechanical energy, which is then converted into electrical energy. This energy generation is termed “pressure retarded” because the process doesn't allow complete equilibrium (which would occur in normal osmosis) but instead harnesses the pressure differential for energy before the system reaches equilibrium. PRO can be used alongside other energy recovery systems in desalination plants to optimise efficiency further [5]. Furthermore, PRO technology can present potentials for reducing SEC in hybrid desalination processes. Ref. [6] highlights the advancements and challenges of integrating PRO with Seawater-RO for sustainable desalination. The study confirms the potential of PRO hybrid systems to reduce energy consumption by up to 20 % and improve desalination efficiency by utilizing osmotic energy from RO brine. It emphasizes advancements in membrane technology, economic feasibility analyses, and optimised configurations for energy recovery. Despite technical challenges like fouling, scaling, and internal concentration polarization, the system demonstrates promise for energy-efficient desalination. Site-specific conditions and supportive policies are crucial for widespread adoption and sustainable freshwater solutions. Ref. [7] investigates the potential of advanced spacer designs in PRO systems to enhance power density and reduce SEC in hybrid seawater reverse osmosis desalination. The study highlights intrinsic limitations, such as internal concentration polarization, and explores the impact of operating parameters and membrane properties. The study emphasizes prioritizing mixing on the draw side due to its larger impact on transmembrane osmotic pressure. Sensitivity analyses reveal that improvements in SEC are limited (<0.9 %), with internal concentration polarization being the dominant constraint. However, advancements in PRO membrane designs could amplify the benefits of advanced spacers, offering a pathway to more energy-efficient desalination.

Forward osmosis (FO) is another emerging hybrid technology that optimises the energy required by RO plants. This technology is more promising for seawater desalination. It utilises the natural process of osmosis, where water moves across a semipermeable membrane from a lower-concentration solution (feed solution) to a higher-concentration solution (draw solution) without requiring significant external energy input. When seawater (feed solution) and the draw solution are placed on opposite sides of the semipermeable membrane, water molecules from the seawater naturally migrate through the membrane into the draw solution due to the osmotic pressure gradient. Salts and other impurities are retained on the feed side. As water enters the draw solution, it becomes diluted. This diluted solution now contains both the draw solutes and the pure water from the seawater. The draw solution must undergo a secondary process, such as reverse osmosis, membrane distillation, or another separation technique to recover fresh water. This step separates the draw solutes from the water, yielding fresh water. FO typically requires less energy than traditional desalination methods like RO as it does not rely on high-pressure pumps [5].

This work investigates the techno-economic impact of three different ERDs on the proposed AWE-driven RO system. The studied ERDs are the Pelton turbine (PL), Pressure Exchanger (PX), and Hydraulic Turbine Charger (HTC) [8,9]. The proposed system in this study is a 2 MW RO system driven by a pumping-mode airborne wind energy system. Pumping-mode AWE has been chosen since it is the most promising and pioneering AWE technology in commercialisation. The same techno-economic studies have been carried out in this article for equivalent wind and solar energy-based RO systems. The study shows that by 2030, when AWE technology is predicted to become fully commercial, the levelised cost of water (LCOW) of an AWE-based RO can be competitive with wind and solar-driven RO systems.

AWE technology has the potential to present a better economic case than conventional wind energy devices due to a 90 % reduction in required civil and mechanical infrastructures [10]. This novel wind energy technology can harness more robust and consistent winds at high altitudes unreachable for HAWTs. However, compared to horizontal axis wind turbines (HAWT), AWE is yet to become a mature technology, and its levelised cost of energy (LCoE) is still high. AWE systems can be classified into two major types: fly-gen and ground-gen. The ground-gen technology, also called pumping mode AWE, uses a kite or glider as a wing tethered to a ground-based generator. Ground-gen AWE operates in a combined cycle, including the power and recovery phases. During the power phase, the wing flies in a controlled trajectory, providing lift force to rotate the tether drum and generator located at the ground station. When the tether arrives at its maximum length, the system must be switched to the recovery phase to retrieve the kite to its initial tether length before starting the cycle of operation. The combined cycle is called the pumping cycle. The system's power consumption during the recovery phase is a fraction of the generated power, so the total power production in a complete pumping cycle is positive. Fly-gen AWE technology utilises generator(s) installed on the flying wing. The wing is tethered to a ground station, and generated power needs to be despatched to the ground station using a cable(s) embedded in the tether [11,12]. Ref. [13] estimates an LCoE of 120 Euro/MWh for a land-based pumping mode AWE system equipped with a kite (soft wing) and with a capacity factor of 54 %. Ref. [14] has calculated almost the same LCoE of 0.106 ₤/kWh (∼127 Euros/MWh) for the utilisation of pumping mode AWE systems in Northern Ireland. Overall, the LCoE of airborne wind is estimated at more than 100 Euro/MWh [15], which is still higher than the LCoEs reported for conventional wind turbines [16]. Compared to traditional wind energy systems, AWE is a developing technology that operates at more minor power scales, requiring further improvement and cost reduction. For instance, the LCoE of conventional wind turbines has reduced from 142 £/MWh in 2010 and 2011 to about £50/MWh in 2019 [15]. A major cause of AWE systems' high LCoE is the predicted high annual operation cost, primarily due to the frequent replacements of kites and the ageing of the materials used in the kite and tether [14]. There is an ambiguity about the durability of the materials used in the wing and tether and the frequency of the needed kite replacement. With advances in materials, access to more practical test data, and the development of multi-megawatt AWEs, the technology will be able to achieve LCoE values of less than 30 €/MWh by 2030 [15]; this LCoE is even lower than the predicted values for the LCoE of conventional wind energy in 2030. Ref. [17] shows that ground-gen and fly-gen AWE systems can achieve the average LCoE of 25 €/MWh and 30 €/MWh, respectively, with an acceptable capacity factor of 57 %. Fly-gen's higher LCoE is due to the higher operational cost. Despite the higher LCoE, fly-gen systems can harness up to 16/27 (59 %) of the wind power, while ground-gen systems can capture a maximum of 4/27 (15 %) of the wind power [18]. In 1980, Loyd estimated that using a kite as large as a C-5A aircraft makes it possible to generate 6.7 MW of electrical power from a 10 m/s wind velocity [19]. However, researchers predict that the 1–2 MW target is more realistic and achievable for AWE systems by 2030 [20,21]. Loyd, the pioneer of AWE systems, estimated that the technology has the potential to generate power of up to 45 MW from a single machine [19]. However, due to the technical and economic constraints in manufacturing and materials of wing and tether, this target is not achievable, at least in the short term. Currently, the only commercial AWE product available is a 200-kW pumping-mode AWE device designed by SkySails for off-grid applications [22].

This research is one of the first attempts to consider airborne wind energy (AWE) for seawater desalination as a hybrid desalination system. The study focuses on the techno-economic analysis of a reverse osmosis (RO) desalination system that utilises airborne wind energy. This is a novel work to estimate the techno-economic feasibility of RO systems powered by AWE technology in 2030 when AWE technology is expected to be fully commercialised.

## 2 Materials and methods

**Fig. 1:**
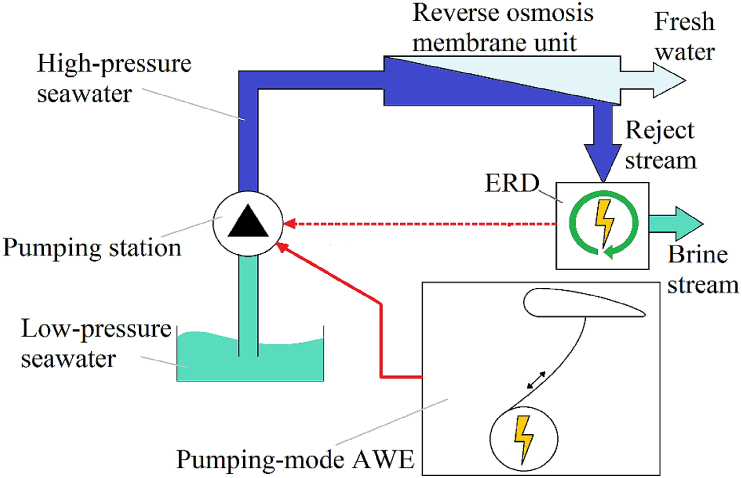
AWE-driven RO system.

The proposed AWE-driven RO is outlined in Fig. 1, and it comprises a pumping station, an RO membrane unit, an energy recovery device, and an AWE system. The pumping station is responsible for pumping seawater into the RO membrane unit with a high pressure of 64 bar. A 2 MW airborne wind energy system supplies the energy for the pumping station. An ERD unit is utilised to retrieve energy from the high-pressure reject stream. The recovered energy helps to improve the efficiency of the electrical energy used at the pumping station and results in higher total water production at the same electrical power consumption at the pumping station.

The techno-economic analysis is based on a 2030 scenario when airborne wind energy systems are expected to be fully commercial. Hence, all the techno-economic parameters and costs used and estimated in this study reflect 2030 values. A 2 MW AWE system is chosen as the size is more realistic and closer to the sizes suggested for commercial AWE systems in 2030 [20,21]. Three ERD technologies have been investigated: Pelton turbine, Pressure Exchanger, and Hydraulic Turbine Charger. Pelton turbine is a centrifugal system widely used in the installed RO plants due to its simplicity and cost-effectiveness. The efficiency of the PL systems can vary between 70 and 80 % [8]. PX is a rotary energy recovery system that uses a rotor to transfer the high pressure of the reject brine stream to the seawater feed stream to achieve higher pressure for the feed seawater stream. They can have efficiencies up to 97 %, a low failure rate, and do not corrode easily [9]. An HTC or turbocharger is a recovery device consisting of a turbine and a pump with a common shaft that uses the high-pressure reject brine to rotate the turbine part, and the compressor part uses the recovered energy to increase the pressure of the seawater stream to feed the pumping station. HTC devices are excellent at controlling the pressure recovery rate and can offer efficiencies of up to 85 % [8,9]. The proposed system has a 2 MWh lithium-ion battery power storage system, which can store and supply the RO's required power for 1 h. The 1-h capacity is chosen because the battery storage system is designed to act only as an emergency backup power supply if a failure to supply power from the AWE system happens. The system's lifetime is 30 years, and a 2 MW pumping mode airborne wind energy system energises the pumping station. Table 1 details the key assumptions and specifications of the presented techno-economic study.

**Table 1:** Techno-economic assumptions and specifications.

**AWE system** [20,23]
*Pumping-mode*
Rated power = 2 MW
CAPEX = 599–956 €/kW (Average = 777.5)
OPEX = 27–54 €/kW/year (Average = 40.5)
LCoE = 33–59 €/MWh (Average = 46)
Capacity factor = 35 %
**Battery Storage** [24]
Battery storage size: 2 MWh
Battery storage CAPEX = 150 €/kWh
Battery OPEX = 10 €/kWh.a
Battery lifecycle = 15 years
**RO system** [8,24,25]; **one unit**
Seawater intake = 2.22 m^3^/h
Water recovery factor = 45 %
Pumping pressure = 64 bar
Capacity factor = 35 %
CAPEX = 2.23 €/(m^3^/year)
OPEX = 4 % of CAPEX
TEC (No ERD) = 150.26 kWh/day
TEC (PL) = 111.83 kWh/day
TEC (PX) = 89.03 kWh/day
TEC(HTC) = 102.96 kWh/day
Specific energy consumption (SEC) = 6.26 kW/m^3^
SEC with Pelton turbine (PL) ERD = 4.66 kW/m^3^
SEC with Pressure Exchanger (PX) ERD = 3.71 kW/m^3^
SEC with Hydraulic Turbine Charger (HTC) ERD = 4.29 kW/m^3^
**Water Supply System** [24]
Pumping station CAPEX = 19.23 €/(m^3^.h.km)
Pumping station OPEX = 2 % of CAPEX
Water pumping distance = 10 m
Pipes CAPEX = 0.053 €/(m^3^.a.km)
Pipes OPEX = 0.023 Euro/(m^3^.a.100 km)
Pipes lengths = 10 m
Water storage capacity: 1-week x daily total water production (m^3^)
Water storage CAPEX = 65 €/m^3^
Water storage OPEX = 2 % of CAPEX
**Wind Turbine** [24]
CAPEX = 1000 €/kW
OPEX = 2 % of CAPEX
LCoE = 60 €/MWh
Capacity factor = 35 %
Lifetime = 30 years
**Photovoltaic (PV) plant** [24]
2 MW Single-axis tracking PV plant
CAPEX = 620 €/kW
OPEX = 1.5 % of CAPEX
LCoE = 60 €/MWh
Capacity factor = 27 % [26]
Lifetime = 30 years
**Other economic parameters** [24,25]
Weighted Average Cost of Capital (WACC) in 2030 = 7 %
Capital recovery factor (CRF)= (WACC∗(1+WACC)^N)/((1+WACC)^N-1) = 8.06 %

A critical factor in designing an RO system is the specific energy consumption (SEC). In this study, the SECs calculated by Ref. [6] are used. The SECs are reported for individual RO units with seawater intake of 2.22 m^3^/h, pumping pressure of 64 bar and water recovery factor of 45 %. Table 1 shows the SECs used in this study. In this study, the input supplied power has a fixed value of 2 MW. This means the focus is on increasing the total water production for the fixed amount of input power to improve the levelised cost of water (LCOW). Based on the SEC and total energy consumption (TEC), the number of individual units that can be utilised by the input supplied power is calculated. Then, considering the number of RO units used, seawater intake, and water recovery factor, the total water production TWP is estimated.

After understanding the TWP of the system based on the ERD system used and the corresponding SEC, it is time to calculate the system's capital expenditure (CAPEX) and operational expenditure (OPEX) using the assumptions and specs mentioned in Table 1. In Table 1, LCoE is the levelised cost of energy and is used to estimate the system's energy cost (EC). Equation (1) can calculate the levelised cost of water (LCOW), where CRF is the capital recovery factor [25].

(1) $$LCOW=\frac{CAPEX\times CRF+OPEX\times EC}{TWP}$$

(2) CAPEX=CAPEX_desal+CAPEX_water+CAPEX_line+CAPEX_res+CAPEX_battery

in (2), *CAPEX_desal* is the capital cost of the desalination plant, *CAPEX_water* is the capital cost of the water supply system, *CAPEX_line* is the CAPEX of the power line for grid connection, *CAPEX_desal* is the CAPEX of the renewable energy source, and *CAPEX_battery* is the capital cost of the battery storage system. Since the study focuses on an off-grid system, the *CAPEX_line* is zero. CAPEX of the water supply system includes the capital cost of water storage, pipes, and the pumping station. The water storage capacity is sufficient to store one week of freshwater production. Due to uncertainties about the cost of AWE systems in 2030, three capital cost scenarios of maximum (AWE_max_), average (AWE_avg_), and minimum (AWE_min_) are investigated. The lifetime of the proposed system is 30 years, while the battery storage can last for 15 years. Hence, *CAPEX_battery* is doubled as the battery storage needs to be replaced with a new one for the second half of the system's lifetime.

Equation (3) calculates the capital recovery factor (CRF), an annuity factor of the desalination system. In (3), WACC is the weighted average cost of capital, and N is the system's lifetime [24].

(3) $$CRF=\frac{{WACC\times \left(1+WACC\right)}^{N}\;}{{\left(1+WACC\right)}^{N}-1}$$

Similar to (2), the OPEX of the system is the sum of the operational costs of the desalination plant, water supply system, renewable energy resources, and battery storage. The predicted maximum, minimum and average LCoEs of airborne wind energy technology in 2030 are used to determine EC. It is assumed that the RO plant does not consume energy from the electrical grid, and the capacity factor of the system is 35 %, i.e., the plant is operational for 3066 h a year. 35 % is chosen as this capacity factor is the most likely capacity factor predicted for AWE systems in 2030. However, capacity factors up to 54 % are also expected for AWE systems in the year 2030 [20].

## 3 Results and analysis

### 3.1 Pumping-mode AWE

**Fig. 2:**
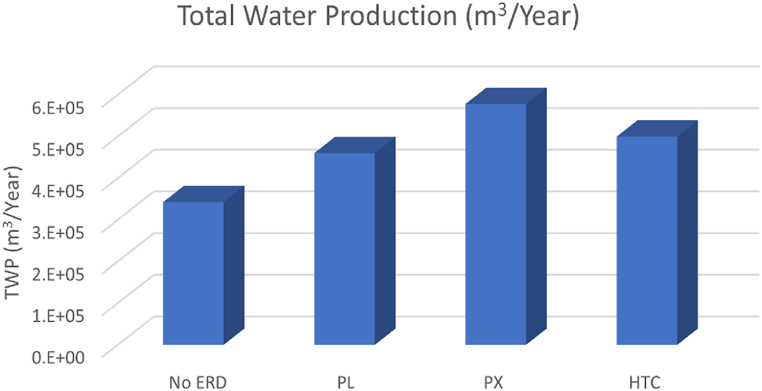
Yearly total water production of AWE-RO system with different ERDs.

Fig. 2 shows the proposed system's yearly total water production (TWP) with different energy recovery devices. The systems' total input power is limited to 2 MW, while the total water production is flexible and can be changed with different SECs and total energy consumption (TEC) values. Considering the TEC of the RO systems with different ERDs and the total input power and the capacity factor of the energy supply system the number of RO units possible to be utilised is calculated and consequently the total water production is estimated.

**Fig. 3:**
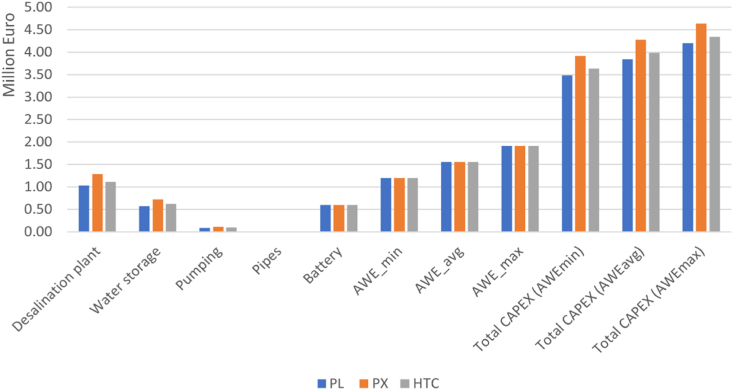
Capital Cost of AWE-RO system for different ERDs.

**Fig. 4:**
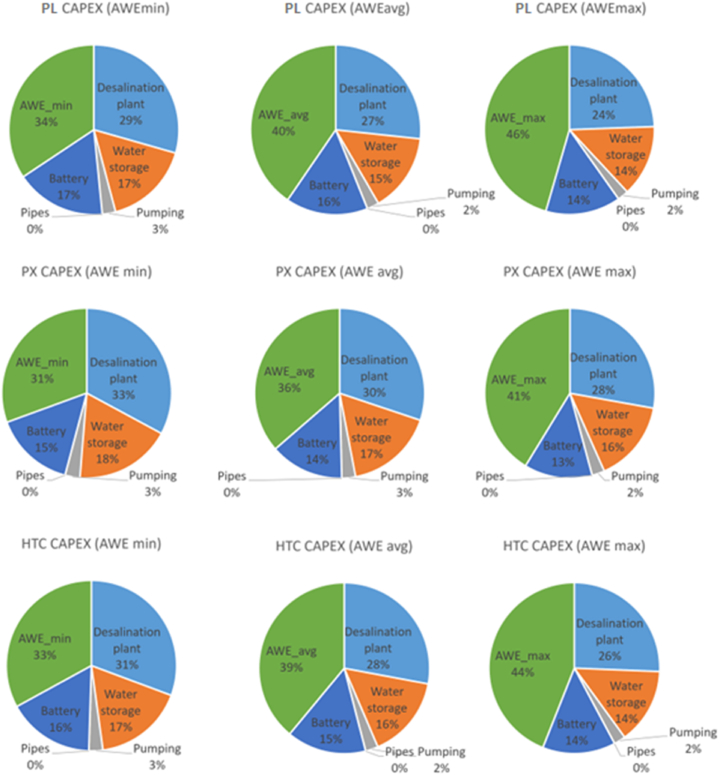
Capital cost breakdown of the proposed AWE-RO in percentage.

The 2 MW AWE-based RO system produces 3.42 million m^3^ of water per year without ERD, while with ERD, the daily TWP increases to 4.6, 5.7, and 5 million m^3^/year for PL, PX, and HTC energy recovery devices, respectively. As can be seen, the PX ERD shows the best TWP with 66 % more freshwater production compared to when no ERD is used. Table 2 and Fig. 3 illustrate the capital cost of the proposed AWE-RO plant. Based on the cost expectations for AWE systems in 2030, three CAPEXs of minimum, average, and maximum have been estimated (see Table 1). The CAPEX calculation investigates the ERDs as well. Depending on the AWE cost scenario and ERD type, the AWE-RO plant's CAPEX can vary from €3.49m to €4.63m. It can be observed that the cost of the AWE-RO plant with pressure exchanger ERD is higher than that of the Pelton turbine and hydraulic turbine charger. Fig. 4 shows the capital cost breakdown with three different energy recovery devices The AWE system needs 31–46 % of CAPEX followed by the desalination plant that uses 24–33 % of the cost. Water storage is the third highest cost in the CAPEX, with the share of 14–18 %.

**Table 2:** CAPEX of the AWE-based reverse osmosis desalination system (M€).

	**PL**	**PX**	**HTC**
Desalination plant	1.03	1.29	1.11
Water storage	0.57	0.72	0.62
Pumping	0.08	0.11	0.09
Pipes	0.000243	0.000306	0.000265
Battery	0.6	0.6	0.6
AWE_min_	1.19	1.19	1.19
AWE_avg_	1.56	1.56	1.56
AWE_max_	1.91	1.91	1.91
Total CAPEX (AWE_min_)	3.49	3.92	3.63
Total CAPEX (AWE_avg_)	3.84	4.28	3.99
Total CAPEX (AWE_max_)	4.20	4.63	4.35

In AWE_min_ scenario, after the cost of the AWE system with share of 31–34 %, the desalination plant possesses the most significant portion of the CAPEX, 29–33 %, followed by the water storage cost, 17–18 %. A similar trend can be seen with the average and maximum cost scenarios of the AWE system. The lithium battery storage costs 13–17 % of the CAPEX. An energy storage system is critical for renewable energy-driven desalination plants. It can improve the system's reliability in dealing with the uncertainty of the RE resources. In this study, the emergency battery storage capacity is enough to reserve and provide energy for the RO plant for 1 h. Larger battery storage capable of supporting the system for a longer time can significantly improve its reliability, although it can be costly and negatively affect the system's economy. The water storage cost at the RO system's output can be even higher than the AWE's. This study assumes one week of water storage capability to ensure a reliable freshwater supply. The pumping station and piping have the lowest portion of the CAPEX, which varies between 2 and 3 % of the total cost. The pumping station is 100 % powered by AWE.

**Fig. 5:**
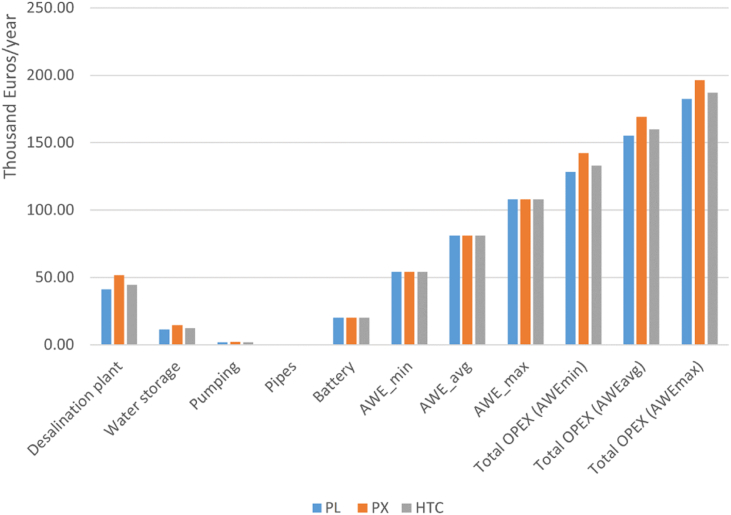
Operating expenditure of AWE-RO system for different ERDs.

**Fig. 6:**
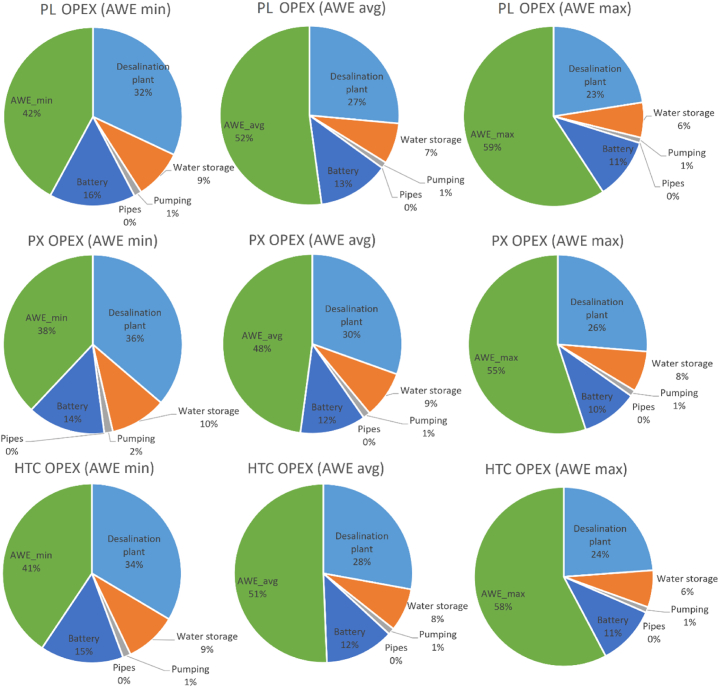
Operational cost breakdown of the proposed AWE-RO in percentage.

A critical factor in the LCOW of freshwater produced by the proposed system is operating expenditure (OPEX), Fig. 5, Fig. 6 illustrates the estimated OPEX of the proposed AWE-powered desalination system. This is carried out based on different AWE cost scenarios (min, avg, max) in 2030 with the three different ERD technologies.

The desalination system with pressure exchanger ERD has the highest OPEX due to its high TWP, followed by HTC and Pelton turbine. For instance, in the AWE_max_ scenario, the PX-based RO system has an annual OPEX of €196,000, while HTC and Pelton-based desalination plants need annual operating costs of €187,000 and €182,000, respectively. If the AWE_min_ scenario is fulfilled in 2030, the yearly operational costs can be decreased to €142,000, €133,000, and €128,000 for PX, HTC, and Pelton turbine-based RO systems, respectively. After the desalination plant, the highest OPEX belongs to the airborne wind energy system. The annual operating cost of the pumping-mode AWE system can be varied between 54,000–108,000 Euros. The Li-ion Batteries require an annual OPEX of €20,000. The water storage system's operational cost differs for ERDs, from €11,500 to €14,400/year. The difference is due to the changes in total water production when each ERD is used. As shown in Fig. 2, the PX-based RO plant has the highest TWP and consequently needs more extensive water storage for the produced fresh water. This also leads to higher CAPEX and OPEX for the RO system with PX ERD technology. Table 3 gives more details about the operational cost of the system.

**Table 3:** OPEX of the AWE-based reverse osmosis desalination system (€/year).

	**Pelton**	**PX**	**HTC**
Desalination plant	4.10E+04	5.16E+04	4.46E+04
Water storage	1.15E+04	1.44E+04	1.25E+04
Pumping	1.77E+03	2.22E+03	1.92E+03
Pipes	1.06	1.33	1.15
Battery	2.00E+04	2.00E+04	2.00E+04
AWE_min_	54000	54000	54000
AWE_avg_	8.10E+04	8.10E+04	8.10E+04
AWE_max_	1.08E+05	1.08E+05	1.08E+05
Total OPEX (AWE_min_)	1.28E+05	1.42E+05	1.33E+05
Total OPEX (AWE_avg_)	1.55E+05	1.69E+05	1.60E+05
Total OPEX (AWE_max_)	1.82E+05	1.96E+05	1.87E+05

Fig. 6 illustrates that the desalination plant's OPEX covers 23–36 % of the system's operational cost. AWE system's OPEX may vary between 38 and 59 % of the total operational cost. The battery storage's OPEX is more consistent; still, it can be between 10 and 16 % of the overall OPEX depending on changes in the total operational cost, and the water storage and pumping station's OPEX are 6–9% and 1 % of the operational cost, respectively.

**Fig. 7:**
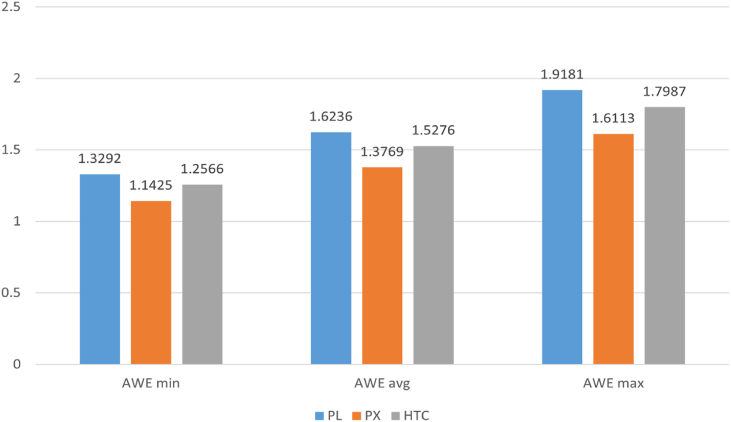
LCOW of AWE-powered RO system with different ERDs (€/m^3^).

Fig. 7 presents the LCOW for the proposed system. The best LCOW has been achieved when the pressure exchanger energy recovery device is used. However, the PX-based RO system poses the highest CAPEX and OPEX. A better total water production (TWP) rate makes it more economical over the 30-year lifetime. The LCOW of the proposed AWE-powered RO system varies between 1.14 and 1.91 €/m^3^ for the different cost scenarios with PX ERD in 2030. The LCOW of the RO system with ERDs of HTC and PL are 1.25–1.79 €/m^3^ and 1.32–1.91 €/m^3^, respectively. The results show a promising LCOW of less than 2 €/m^3^ (1.91) for the most expensive AWE cost scenario in 2030. However, it is essential to understand how much this cost would be comparable to similar RO systems powered by other renewable resources. This will be investigated later in this article.

### 3.2 AWE-based RO vs. wind and solar-based RO

This section investigates the techno-economic study of two similar 2 MW RO systems, one powered by a conventional horizontal axis wind turbine (HAWT) and another powered by photovoltaic (PV) solar panels. Finally, the results are analysed to evaluate the techno-economic viability of the proposed AWE-based RO system. The HAWT and PV-based RO studies reflect the global cost values in 2030; Hence, their techno-economic results reflect the costs of the RO systems in 2030. Parameters and assumptions are reported in Table 2.

**Fig. 8:**
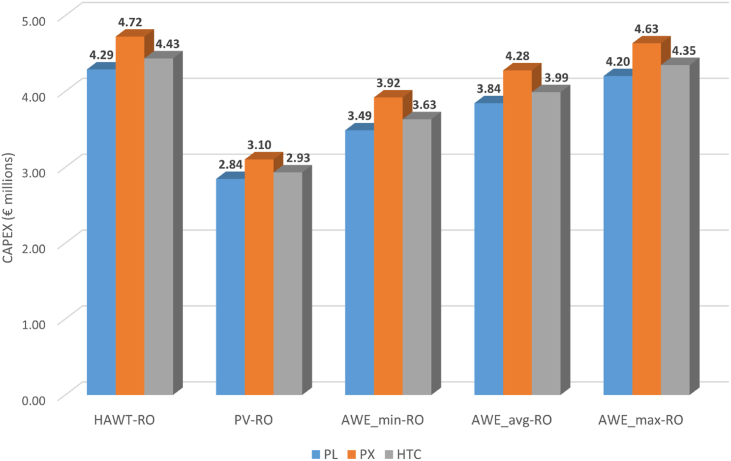
CAPEX of the RE-based RO system with different REs and ERDs (M€).

The proposed reverse-osmosis desalination system powered by airborne wind energy has shown promising results compared to HAWT and PV-based RO systems. In Fig. 8, AWE-RO's capital cost is highly competitive against the HAWT-based system. Even the maximum cost scenario for airborne wind energy in 2030 offers CAPEXs slightly lower (about 2 %) than the HAWT-RO system. The changes are more considerable if the average and minimum cost scenarios come through in 2030. The AWE_min_-RO's CAPEX is 3.49–3.92 million Euros, about 20 % less than the HAWT-RO. For the AWE's average cost scenario in 2030, the capital cost of the AWE-RO is about 10 % cheaper than the HAWT-RO system. The results show that the CAPEX of the PV-powered RO system will be the most challenging rival for the proposed AWE-RO system in 2030. As can be seen, all three AWE cost scenarios present CAPEXs notably higher than the PV-based RO system. For the AWE_min_, AWE_max_, and AWE_avg_ scenarios, the capital expenditure of the proposed AWE-RO systems is 22–26 %, 35–38 %, and 47–49 % more expensive compared to the PV-based RO, respectively. There is a significant difference between the CAPEX of the RO plant with PV and AWE_min_, although the PV's CAPEX is slightly higher (3.5 %) than the AWE_min_ CAPEX (see Table 2). The critical factor here is the capacity factor. The lower capacity factor of the PV system decreases the total water production of the system and, consequently, the size of the desalination plant and water storage system, which are crucial in the total CAPEX of the RO system.

**Fig. 9:**
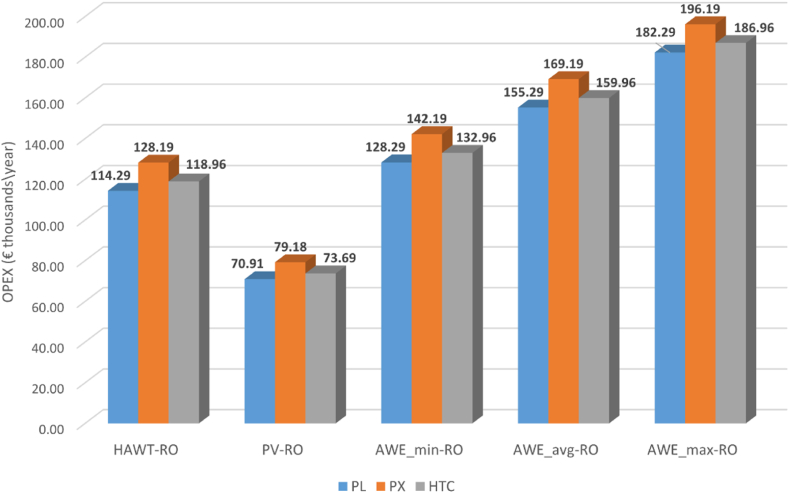
OPEX of the RE-based RO system with different REs and ERDs (x 1000 €/year).

Fig. 9 compares the operating cost of the proposed AWE-RO system with the PV-RO and HAWT-RO systems. As can be seen, the OPEX of the AWE-RO is higher than both HAWT and PV-based ROs. This can be the most critical challenge in dealing with AWE for reverse-osmosis desalination. Depending on the ERD technology used, the AWE_max_ scenario's OPEX varies between 182,290–196,190 €/year. This is 53–59 % and 147–157 % higher than the operating cost needed for HAWT-RO and PV-RO, respectively. For the average cost scenario, the AWE-RO's OPEX varies between 31 % and 35 % higher than HAWT-RO. AWE_avg_ scenario's operating cost is 113–118 % more expensive than PV-RO. Even if the most optimistic cost scenario AWE_min_ fulfils in 2030, the OPEX of the AWE-RO is still 10–12 % and 78–80 % higher than the HAWT-RO and PV-RO, respectively.

**Fig. 10:**
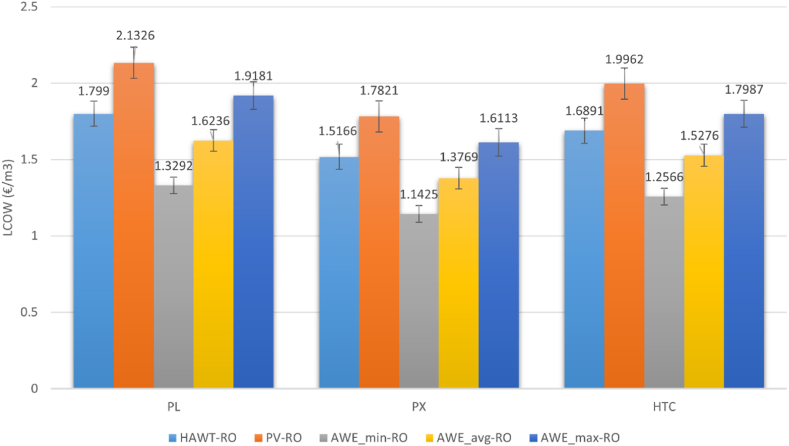
LCOW of the RE-based RO systems with different REs and ERDs (€/m^3^).

The study shows that the LCOW can be competitive for the proposed AWE-RO compared to the other RE-based reverse-osmosis systems. Fig. 10 investigates the LCOW of the AWE-RO system against HAWT and PV-based ROs. The best LCOW is 1.14 €/m^3^, achieved for the AWE_min_ scenario when a pressure exchanger energy recovery device is utilised. This LCOW is even lower than the LCOW of the PV-RO, where the levelised cost of water is 1.78 €/m^3^ with a similar ERD technology. For the AWE_avg_ scenario, the LCOW of the AWE-RO is still lower than the PV-RO's and HAWT-RO's LCOW. However, the results for the AWE_max_ scenario show that the best LCOW for AWE-RO is 1.61 €/m^3^, which is higher than the LCOW of HAWT-RO but still lower than the PV-RO. The best LCOW is achieved when the pressure exchanger ERD is used. Using a hydraulic turbine charger presents higher LCOW, while the most expensive LCOW is achieved when the Pelton turbine is used. The highest LCOW is 2.13 €/m^3^, which belongs to the PV-RO with a Pelton turbine energy recovery device. Higher capacity factor and TWP assist AWR-RO in presenting better LCOW compared to the PV-RO in AWEmax, AWEmin and AWE_avg_ scenarios. At the same time, CAPEX and OPEX are higher than PV-RO in all the scenarios.

## 4 Discussion and conclusions

The study investigates the techno-economic feasibility of an off-grid reverse-osmosis system powered by AWE technology. This research aims to evaluate the techno-economic performance of an AWE-RO system compared to the widely used renewable energy technologies, including solar and wind energy. As AWE systems won't be fully commercialised by 2030, the study is carried out in the context of 2030. A range of AWE cost estimations are considered, and three scenarios of AWE_min_, AWE_avg_, and AWE_max_ are implemented for the techno-economic study. The study also investigates various energy recovery devices, including Pelton turbine, hydraulic turbine charger, and pressure exchanger.

In the AWE_max_ scenario, it is estimated that the 2 MW AWE system will need a capital cost of €1.9m. On the other hand, the AWE_min_ scenario needs a CAPEX of about €1.2m, while the AWE_avg_'s capital cost is €1.56m. The CAPEX of the whole AWE-based RO system is estimated between 3.49-€4.63m, depending on the AWE scenario and the ERD technology. In all scenarios, including the AWE_max_, this CAPEX is less than that needed for the RO system powered by conventional wind turbines. However, compared to where solar photovoltaic is utilised for the RO system, the proposed AWE-powered RO presents higher CAPEX in the AWE_min_, AWE_avg_, and AWE_max_ scenarios and, therefore, can be concluded that the AWE_RO system is more expensive than PV-RO in terms of capital cost.

The AWE-RO system with pressure exchanger ERD presents the highest CAPEX and OPEX. However, due to its significant contribution to increasing the total water production and reducing specific energy consumption, it achieves the lowest LCOW. The best LCOW for the proposed AWE-RO is 1.14 €/m^3^. This LCOW is achieved with a PX ERD when the AWE_min_ scenario is applied. This is lower than the LCOW estimated for PV-RO and HAWT-RO with any ERD. Nevertheless, for the AWE_max_ scenario, the LCOWs of the proposed system are more expensive than HAWT-RO. In AWE_avg,_ the estimated LCOWs are less than the LCOWs calculated for HWAT-RO and PV-RO.

Overall, the results show that in AWE_min_ and AWE_avg_ scenarios, the proposed AWE-powered RO can be highly competitive against the other RE-based RO systems. However, the AWEmax scenario has been found to have an inferior techno-economic performance compared to HAWT-RO. A potential weak point of the proposed AWE-RO system is its high OPEX. The study indicates that the AWE-RO incurs higher OPEX than PV-RO and HAWT-RO in all scenarios. AWE-RO with PX ERD needs the highest OPEX compared to the other RO systems. For instance, the OPEX of the AWE-RO with PX in the AWE_max_ scenario is about 148 % higher than the OPEX of the PV-RO with a similar ERD system.

The study shows the significant role of energy cost on the techno-economy of the RO plant. Different renewable energy sources and scenarios have been evaluated confirming that the energy cost plays the most important parameter in the economy of the RO plant. In addition, the research work shows SEC can significantly impact the economy of reverse osmosis (RO) power plants, as it directly influences operational costs, overall profitability and freshwater production. Lower SEC reduces the energy required to produce freshwater, thereby minimising energy cost, which constitute a major share of operating costs in RO plants. It can also enable compliance with environmental regulations, enhances sustainability, and ensures economic feasibility in energy-scarce regions. Additionally, efficient energy use reduces energy price and justifies investments in advanced technologies. For large-scale plants, even minor SEC reductions yield significant savings, supporting the importance of energy efficiency for the profitability and competitiveness of RO desalination projects.

The study confirms the techno-economic feasibility of using AWE for reverse-osmosis seawater desalination to pave the way for future research on AWE-RO systems. The size of the water reservoir and energy storage system can critically influence the system's techno-economic model. Moreover, to enhance the proposed AWE-RO system's techno-economic model, advanced techno-economic models, optimisation, incorporating different renewable energy systems with the airborne wind energy-RO, and power grid integration can be investigated in future works.

## CRediT authorship contribution statement

**Mahdi Ebrahimi Salari:** Writing – review & editing, Writing – original draft, Visualization, Validation, Software, Resources, Methodology, Investigation, Formal analysis, Data curation, Conceptualization. **Milad Zabihi:** Writing – review & editing, Validation, Methodology, Investigation, Formal analysis. **Jimmy Murphy:** Writing – review & editing, Supervision, Resources, Project administration, Methodology, Funding acquisition.

## Data availability statement

All the research data can be made available upon request. Please contact the corresponding author to access the data.

## Declaration of competing interest

The authors declare that they have no known competing financial interests or personal relationships that could have appeared to influence the work reported in this paper.

## References

[1] Kalogirou S.A. (2005). Seawater desalination using renewable energy sources. Prog. Energy Combust. Sci..

[2] Thi D., Trang H., Pasztor T., Fozer D., Manenti F., Toth A.J. (2021). Comparison of desalination technologies using renewable energy sources with life cycle, PESTLE, and multi-criteria decision analyses. Water.

[3] Panagopoulos A. (2021). Water-energy nexus: desalination technologies and renewable energy sources. Environ. Sci. Pollut. Control Ser..

[4] Eltawil M.A., Zhengming Z., Yuan L. (2009). A review of renewable energy technologies integrated with desalination systems. Renew. Sustain. Energy Rev..

[5] Harby K., Emad M., Benghanem M., Abolibda T.Z., Almohammadi K., Aljabri A., Alsaiari A., Elgendi M. (2024). Reverse osmosis hybridization with other desalination techniques: an. Desalination.

[6] Ahmed A., Alghamdi A., Ahmed S. (2024). Techno-economic analysis of a pressure retarded osmosis (PRO) - seawater reverse osmosis (SWRO) hybrid: a case study. Front. Energy Res..

[7] Ng W., Liang Y., Weihs G.F. (2023). Quantifying the potential of pressure retarded osmosis advanced spacers for reducing specific energy consumption in hybrid desalination. J. Water Proc. Eng..

[8] Gude V.G. (2012). Energy consumption and recovery in reverse osmosis. Desalination Water Treat..

[9] Huang B., Pu K., Wu P., Wu D., Leng J. (2020). Design, selection and application of energy recovery device in seawater desalination: a review. Energies.

[10] BVG Associates (September 2022). https://airbornewindeurope.org/studies-papers/white-paper-for-the-airborne-wind-energy-sector-by-bvg-associates-commissioned-by-airborne-wind-europe/.

[11] Cherubini A., Papini A., Vertechy R., Fontana M. (2015). Airborne wind energy systems: a review of the technologies. Renew. Sustain. Energy Rev..

[12] Pereira A.F.C., Sousa J.M.M. (2023). A review on crosswind airborne wind energy systems: key factors for a design choice. Energies.

[13] Faggiani P., Schmehl R. (2018). Airborne Wind Energy: Advances in Technology Development and Research.

[14] Lunney E., Ban M., Duic N., Foley A. (2017). A state-of-the-art review and feasibility analysis of high-altitude wind power in Northern Ireland. Renew. Sustain. Energy Rev..

[15] Mann S. (February 2019). https://ore.catapult.org.uk/wp-content/uploads/2019/02/An-Introduction-to-Airborne-Wind-Stephanie-Mann-AP0020.pdf.

[16] Bosch J., Staffell I., Hawkes A. (2019). Global levelised cost of electricity from offshore wind. Energy.

[17] Trevis F., McWilliam M., Gaunaa M. (2021). Configuration optimisation and global sensitivity analysis of ground-gen and fly-gen airborne wind energy systems. Renew. Energy.

[18] Lellis M.D., Reginatto R., Saraiva R., Trofino A. (2018). The betz limit applied to airborne wind energy. Renew. Energy.

[19] Loyd M.L. (1980). Crosswind kite power. J. Energy.

[20] Weber J., Marquis A.C.M., Draxl C., Hammond R., Jonkman J., Lemke A., Lopez A. (August 2021). https://www.nrel.gov/docs/fy21osti/79992.pdf.

[21] Weber J., Marquis M., Lemke A., Cooperman A., Draxl C., Lopez A., Roberts O., Shields M. (July 2021). https://www.nrel.gov/docs/fy21osti/80017.pdf.

[22] Skysails (2022). Onshore unit AKS PN-14 - a revolution for onshore wind power. https://skysails-power.com/onshore-unit-pn-14/.

[23] Heilmann J. (2012). https://studenttheses.uu.nl/handle/20.500.12932/12243.

[24] Caldera U., Bogdanov D., Breyer C. (2016). Local cost of seawater RO desalination based on solar PV and wind energy: a global estimate. Desalination.

[25] Azinheira G., Segurado R., Costa M. (2019). Is renewable energy-powered desalination a viable solution for water stressed regions? A case study in algarve, Portugal. Energies.

[26] Boretti S.C.A. (2020). Trends in performance factors of large photovoltaic solar plants. J. Energy Storage.

